# Diluted Etilefrine for Neonatal Chylothorax: A 96-Hour Physicochemical Stability Study

**DOI:** 10.3390/pharmaceutics18091135

**Published:** 2026-09-09

**Authors:** Louisa Bourel, Sixtine Gilliot, Zeina Boulos, Yousra Alouadni, Kévin Le Duc, Mohamed Riadh Boukhris, Laurent Storme, Anthony Martin Mena, Pascal Odou

**Affiliations:** 1 CHU Lille, ULR 7365-GRITA-Groupe de Recherche sur les Formes Injectables et les Technologies Associées, University Lille, F-59000 Lille, Franceyousra.alouadni.etu@univ-lille.fr (Y.A.);; 2 CHU Lille, ULR 2694 METRICS: Evaluation des Technologies de Santé et des Pratiques Médicales-Perinatal Environment and Health, University Lille, F-59000 Lille, France; 3 CHU Lille, Department of Neonatology, Jeanne de Flandre Hospital, F-59000 Lille, France; riadh.boukhris@chu-lille.fr

**Keywords:** etilefrine, drug stability, chylothorax, neonatal intensive care

## Abstract

**Background/Objectives:** Etilefrine is a sympathomimetic agent increasingly used off-label as a rescue therapy for refractory neonatal chylothorax. For continuous intravenous administration in neonates, the commercially available 10 mg/mL formulation requires substantial dilution to a concentration of 4 µg/mL. However, stability data for these highly diluted preparations are currently lacking. Hence, this study aimed to assess the short-term physicochemical stability of etilefrine diluted to 4 µg/mL in 0.9% sodium chloride or 5% dextrose. **Methods:** Etilefrine was quantified using a high-performance liquid chromatography method coupled with ultraviolet detection. Diluted solutions were prepared in 10 mL polypropylene syringes, maintained at room temperature, and analyzed daily for 96 h. Physicochemical stability assessment included visual inspection for physical changes, assays of drug concentration, pH, osmolality, and subvisible particle counts. **Results:** Throughout the 96 h, no visible changes, such as precipitation, discoloration, or haze, were observed. Etilefrine concentrations in both isotonic diluents remained within 10% of the initial value. There were no significant variations in pH and osmolality. Subvisible particle counts remained below the limits established by the European Pharmacopoeia. **Conclusions:** Etilefrine diluted to 4 µg/mL in 0.9% sodium chloride or 5% dextrose in syringes remained physicochemically stable for 96 h at room temperature. These findings provide practical stability data to support the preparation and administration of diluted etilefrine, and may facilitate the standardization of its use in future clinical studies of refractory neonatal chylothorax.

## 1. Introduction

Chylothorax in neonates is characterized by the accumulation of chyle within the pleural space between the lungs and the thoracic cage [1,2]. It can lead to lung underdevelopment, heart failure secondary to compromised vascular flow, malnutrition, increased susceptibility to infections and bleeding complications [3]. Neonatal chylothorax may be congenital (1 in 10,000 to 24,000 births, with a mortality rate of approximately 28%), traumatic, or non-traumatic [3,4]. Severe chylothorax requires thoracentesis to drain chyle and relieve lung compression, alongside supporting ventilation and oxygenation [4,5]. Newborns are placed on complete bowel rest with total parenteral nutrition. If conservative measures are insufficient, pharmacological treatments, primarily somatostatin and its analogs, may be employed as they reduce splanchnic blood flow and gastrointestinal secretions [2]. However, their use is limited due to the potential risk of necrotizing enterocolitis [6]. Invasive interventions, such as thoracic duct ligation or embolization, are technically complex and risky in low-birth-weight preterm infants [2,4,5]. Consequently, there is a critical need for pharmacological options in refractory chylothorax.

Etilefrine, a sympathomimetic agent originally employed to treat orthostatic hypotension and priapism, has recently emerged as a potential rescue therapy for life-threatening neonatal chylothorax [7,8,9,10,11]. The mechanism of action of etilefrine in chylothorax remains unclear. It is thought to stimulate alpha and beta adrenoreceptors, causing contraction of smooth muscle fibers in the thoracic duct, reducing its diameter and chyle flow, thereby promoting leak closure and healing [8,9].

In our 2025 case series, five newborn infants with chylothorax experienced a marked reduction in chest-tube drainage within 2–4 days after initiation of continuous intravenous etilefrine, while heart rate and blood pressure remained within age-appropriate ranges [9]. Nevertheless, this observational experience does not establish efficacy, and controlled studies are required. Furthermore, the practical aspects of etilefrine administration in neonates introduce additional challenges that must be addressed.

Specifically, cases reported in neonates described the administration of etilefrine as a highly diluted solution at low infusion rates ranging from 0.2 to 1 µg/kg/h [8,9,10]. Such administration requires substantial dilution of the commercial 10 mg/mL formulation to 4 µg/mL using either 0.9% sodium chloride (NaCl) or 5% dextrose in water (D5W). These dilutions are prepared bedside by trained nurses, and syringes are infused over several hours. In the absence of physicochemical stability data for these diluted solutions, the syringe replacement intervals are empirical. Investigating the stability of diluted etilefrine could optimize syringe replacement, reduce drug wastage, and limit manipulation of intravenous lines in this vulnerable population. Furthermore, as etilefrine hydrochloride contains no excipients other than water for injection, it could be potentially sensitive to extensive dilution or diluent changes. Ensuring the stability of diluted etilefrine is crucial for accurate drug delivery and to support its safe use in future clinical studies, especially given the life-threatening nature of refractory neonatal chylothorax.

Therefore, this study aimed to assess the physicochemical stability of etilefrine diluted to 4 µg/mL in 0.9% NaCl and D5W, stored at room temperature in polypropylene (PP) syringes (10 mL and 50 mL) over 96 h.

## 2. Materials and Methods

### 2.1. Chemicals

The etilefrine used in this study was from a 10 mg/mL ampoule (1 mL) produced by Serb Pharmaceuticals (Paris, France), containing etilefrine hydrochloride and water for injection as the sole excipient. The commercial solution was directly diluted in either D5W (Baxter, Deerfield, IL, USA) or in 0.9% NaCl (Baxter, Deerfield, IL, USA) to achieve the expected concentrations. All reagents were of analytical grade, and came from VWR International (Radnor, PA, USA), except acetonitrile (ACN) (Carlo Erba, Milan, Italy) and sodium octanesulfonate monohydrate (ThermoScientific, Waltham, MA, USA). Ultrapure water was homemade from a Purelab^®^ purification system (Veolia, ELGA Labwater, Lane End, UK).

### 2.2. Quantification of Etilefrine by High-Performance Liquid Chromatography

#### 2.2.1. Stock Solution Preparation

Calibration solutions were prepared by diluting the commercial 10 mg/mL etilefrine hydrochloride in graduated flasks with D5W or 0.9% NaCl. Seven concentration levels were prepared corresponding to 60%, 80%, 90%, 100%, 110%, 120%, and 140% of the target concentration (2.4, 3.2, 3.6, 4.0, 4.4, 4.8, and 5.6 µg/mL). These solutions were injected into the high-performance liquid chromatography (HPLC) system without additional dilution.

#### 2.2.2. HPLC System

An HPLC system (Shimadzu, Noisiel, France) was used to quantify etilefrine. It was equipped with a degassing DGU-20A3R unit, two LC-20ADXR solvent delivery units (Prominence UFLCXR series), an SIL-30AC autosampler, a CTO-20AC column oven and an SPD-M20A photodiode array detector (DAD).

#### 2.2.3. Chromatographic Conditions

The mobile phases consisted of ACN and an ammonium formiate buffer mixture in a ratio of 10% to 90%, respectively, under isocratic conditions. The buffer consisted of 236 mg of sodium octanesulfonate monohydrate, 6.4 mL of 99% formic acid, and 11.2 mL of 28% ammonia solution, which were diluted in ultrapure water (qs one liter of buffer) and adjusted to a pH of 3 using hydrochloric acid (HCl). The flow rate was maintained at 0.25 mL/min, and each run lasted 15 min. The separation of etilefrine was carried out on a Luna^®^ Omega PS C18 column (100 × 2.1 mm, 1.6 µm, 100 Å; Phenomenex, Torrance, CA, USA), preceded by a PS C18 cartridge guard (AJ0-9508; Phenomenex, Torrance, CA, USA). The temperature of the column oven was fixed at 40 °C. The injection volume was set at 10 µL, and quantification was performed at 273 nm.

#### 2.2.4. Degradation Study to Validate the Stability-Indicating HPLC Method

The degradation protocol complied with the SFPC GERPAC methodological guidelines. Forced degradation conditions are presented in Table 1, in accordance with the stress condition limits set by the SFPC GERPAC guidelines [12]. Etilefrine was exposed to the tested extreme conditions until approximately 20% degradation of its initial concentration was reached. Subsequently, samples were analyzed using HPLC-DAD. The selectivity of the method was based on comparing the chromatogram of the quality control etilefrine solution with the chromatograms obtained from the degradation study. For each degradation protocol, samples were at the target concentration of 4 µg/mL prior to injection into the HPLC system.

For the photodegradation study, samples were exposed for 48 h to UVA and visible light using a BINDER KBF P 240 controlled-climate chamber equipped with an ICH Q1B light module (BINDER GmbH, Tuttlingen, Germany). According to the manufacturer’s specification, the UVA source covers the 320–400 nm spectral range, while the visible-light source covers the visible spectral range. The chamber temperature was controlled at 20 °C throughout the irradiation period.

### 2.3. Preparation and Storage of Etilefrine Syringes

Etilefrine was sequentially diluted using volumetric flasks to ensure precision, reducing its concentration from 10 mg/mL to 4 µg/mL. Initially, a 1:500 dilution was prepared, followed by a 1:5 dilution. The solvents employed were D5W or 0.9% NaCl. Subsequently, 10 mL of 4 µg/mL etilefrine was manually transferred to 10 mL polypropylene (PP) Luer-lock syringes (BD Plastipak, Franklin Lakes, NJ, USA), or 50 mL was transferred in 50 mL PP Luer-lock syringes (BD Plastipak, Franklin Lakes, NJ, USA). For each dilution condition (D5W and 0.9% NaCl), 24 syringes of 10 mL and 12 syringes of 50 mL were prepared to meet the volume requirements for the different analyses. The syringes were stored at controlled room temperature (20 ± 2 °C) for 96 h, unprotected from light.

### 2.4. Stability Study Design

Stability of etilefrine 4 µg/mL in D5W and 0.9% NaCl was assessed at different times (hours): T0, T24 h, T48 h, T72 h, and T96 h. The same three syringes were used for all the following analyses at each sampling point. Initially, the three syringes were examined macroscopically against a black and white background, using unaided vision, to observe the presence of precipitation, crystallization, color change, or haze daily. Then, etilefrine concentration was determined using the HPLC-DAD validated method described above. To assess chemical stability, etilefrine concentration was expressed as the ratio, in percentage, of the concentration measured at sampling times to the initial concentration in the syringe (Ct/C0, %). The solution was considered stable if the concentration remained above 90% of C0 [13]. Subsequently, the osmolality was measured using a 3300 Micro-Osmometer (Advanced Instruments, Norwood, MA, USA), and the pH was monitored using a pH microelectrode HI1083 (Hannah Instrument, Woonsocket, RI, USA). These two parameters were considered stable if no significant changes were detected. Finally, etilefrine solutions in syringes were analyzed using a particle counting system (APSS-2000, Particle Measuring Systems, Niwot, CO, USA) in accordance with the European Pharmacopoeia Monograph 2.9.19 [14]. At T0 and T96 h, three 10 mL syringes were pooled and tested three times using a light obscuration counter, in triplicate (*n* = 9 measurements on nine syringes). For 50 mL syringes, 30 mL was sampled in three syringes and analyzed three times separately at T0 and T96 h (*n* = 9 measurements on 3 syringes). To maintain physicochemical stability, the number of particles ≥10 µm and ≥25 µm should not exceed 6000 and 600 particles per syringe, respectively.

### 2.5. Statistical Analysis

Calibration ranges were established by plotting the peak areas of etilefrine hydrochloride calibration solutions, diluted in D5W or 0.9% NaCl, against their respective etilefrine concentrations. Each calibration curve consisted of seven concentration levels. Three independent calibration ranges were prepared and analyzed each day over three consecutive days, resulting in nine calibration curves for each diluent. Linearity of the method was assessed by analysis of variance (ANOVA) for the lack-of-fit at the 95% confidence level, preceded by a Cochran test to verify the homogeneity of variances. Method validation was subsequently conducted using the SFSTP accuracy profile approach based on systematic and random errors. All calculations were performed on back-calculated concentrations obtained by applying the linear regression model to the signals of the validation standards [15,16]. Trueness (systematic error) was assessed at each concentration level by calculating the relative bias (%) and the recovery (%), representing the difference between the mean of back-calculated concentrations and the nominal concentration. Precision (random error) was evaluated by estimating repeatability and intermediate precision. Repeatability was estimated as the square root of the intra-series variance, while intermediate precision was calculated as the square root of the sum of intra-series and inter-series variances. The results are expressed as coefficients of variation (CV) relative to the mean calculated concentration for each level. Acceptance criterion for precision was predefined as 5% [16]. Accuracy of the method for the quantification of etilefrine was evaluated using β-expectation tolerance intervals (β = 90%), calculated for each concentration level according to the SFSTP methodology [16]. These intervals combine systematic and random errors to estimate the range within which a future measurement is expected to fall with a probability of 90%. The analytical method was considered valid when the β-expectation tolerance intervals remained within the predefined acceptance limits of ± 10% across the entire calibration range.

The pH values and osmolality results obtained at T0 and T96 h were compared using a paired Wilcoxon signed-rank test (α = 5%) for each condition and storage tested, using R software (Version 1.2.1335; R Foundation for Statistical Computing, Vienna, Austria).

Graphs were generated using GraphPad Prism (version 8.0.2, GraphPad Software, Boston, MA, USA).

## 3. Results

### 3.1. Forced Degradation Study

**Figure 1:**
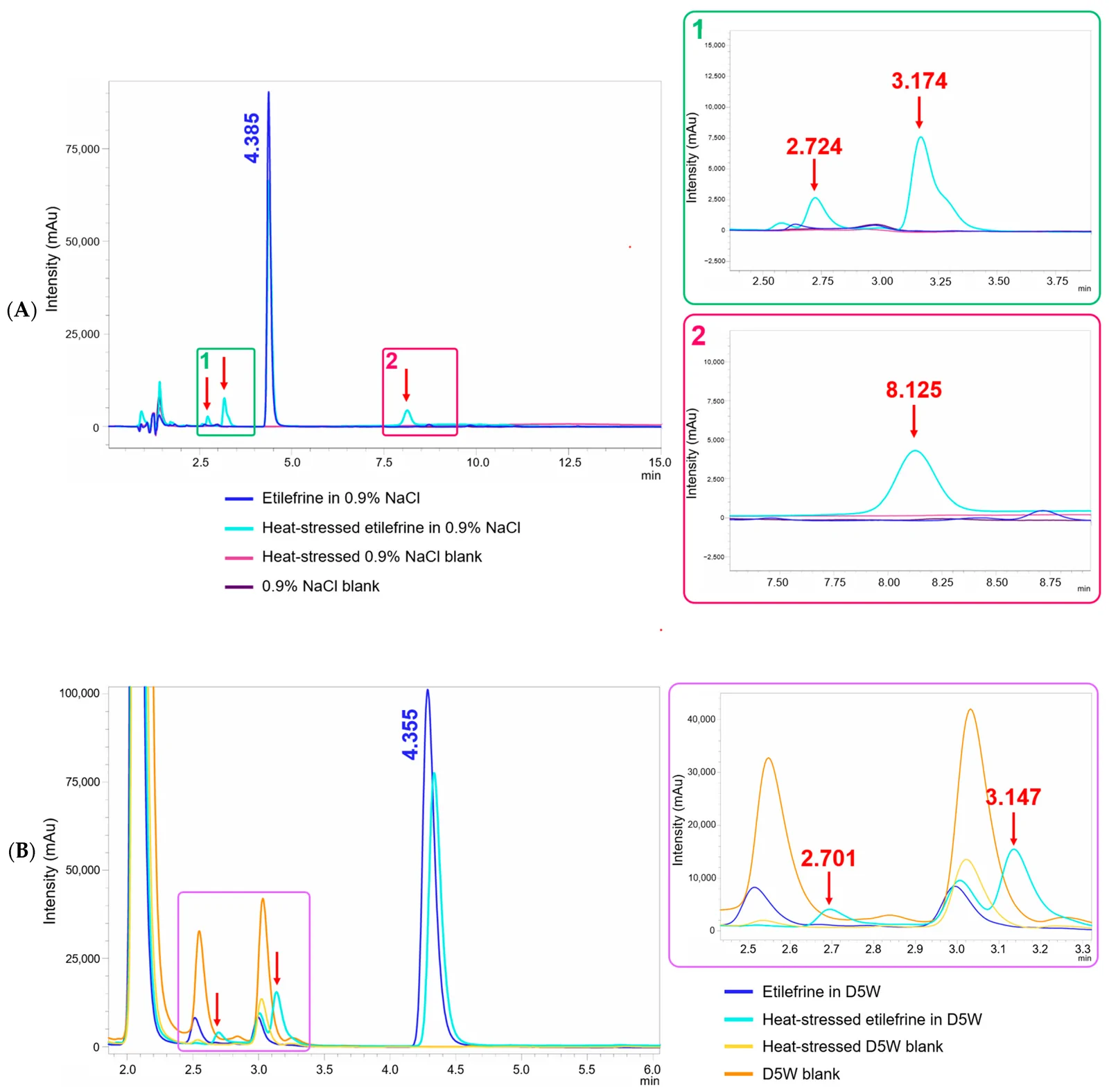
Overlay chromatograms of blanks and etilefrine solutions before and after thermal stress when diluted in (**A**) 0.9% NaCl (80 °C, 48 h) and (**B**) dextrose 5% in water (D5W) (80 °C, 8 h). Peaks of degradation products are marked by a red arrow.

The chromatogram of the stock solution was compared with those obtained after forced degradation. Etilefrine was detected at a retention time (RT) of 4.385 ± 0.010 min and 4.355 ± 0.020 min when diluted in either 0.9% NaCl or D5W (*n* = 3 in each diluent). Heat exposure induced approximately 20% degradation of etilefrine in both diluents (Figure 1). Two degradation peaks were consistently observed under both conditions (RT 2.724 ± 0.004 and 3.174 ± 0.006 min in 0.9% NaCl; RT: 2.701 ± 0.006 and 3.147 ± 0.010 min in D5W). An additional degradation peak was detected only in 0.9% NaCl (RT 8.125 ± 0.005 min). Under all other degradation conditions in both diluents, no degradation peaks were detected, and etilefrine remained largely intact (less than 1% reduction). The method was highly selective, with no interference between the molecule and degradation products.

### 3.2. Validation of the HPLC-DAD Method over the 2.4–5.6 µg/mL Concentration Range for Etilefrine

Linearity was established within the concentration range of 2.4 to 5.6 µg/mL for both diluents. The Cochran test indicated homogeneous variances across the seven concentration levels, with calculated values of 0.3135 for 0.9% NaCl and 0.2677 for D5W, both of which were below the critical value of 0.3535. The ANOVA lack-of-fit test did not reveal significant deviation from linearity, yielding *p*-values of 0.79 for 0.9% NaCl and 0.965 for D5W. The calibration curves exhibited determination coefficients (r^2^) of 0.9986 for 0.9% NaCl and 0.9961 for D5W. The y-intercepts were not significantly different from zero in either diluent (*p* > 0.05). These results confirm the suitability of a linear regression model over the investigated calibration range. The limits of detection (LOD) and quantification (LOQ) were determined to be 0.079 and 0.158 µg/mL for 0.9% NaCl, and 0.131 and 0.262 µg/mL for D5W, respectively. The validation results demonstrated satisfactory trueness and precision over the entire calibration range for both diluents. In 0.9% NaCl, the relative bias ranged from −0.39% to 0.35% and the CV for repeatability from 0.59% to 0.89%, while the CV for intermediate precision was between 0.84% to 1.10%. In D5W, the relative bias ranged from −0.32% to 0.33%, the CV for repeatability from 0.71% to 1.44%, and the CV for intermediate precision from 1.45% to 1.80%. All precision values remained below the predefined acceptance criterion of 5%, and all β-expectation tolerance intervals were contained within the predefined acceptance limits of ±10%, confirming the accuracy of the analytical method throughout the calibration range (see Supplementary Material).

### 3.3. Stability Assessment of Etilefrine 4 µg/mL for 96 h

#### 3.3.1. Macroscopic Analysis

Over the 96-h duration of the stability study, no observable alterations were detected in the diluted etilefrine solutions. Specifically, there were no visible particles, no changes in coloration, and no haze detected at any point during this period.

#### 3.3.2. Chemical Stability

**Figure 2:**
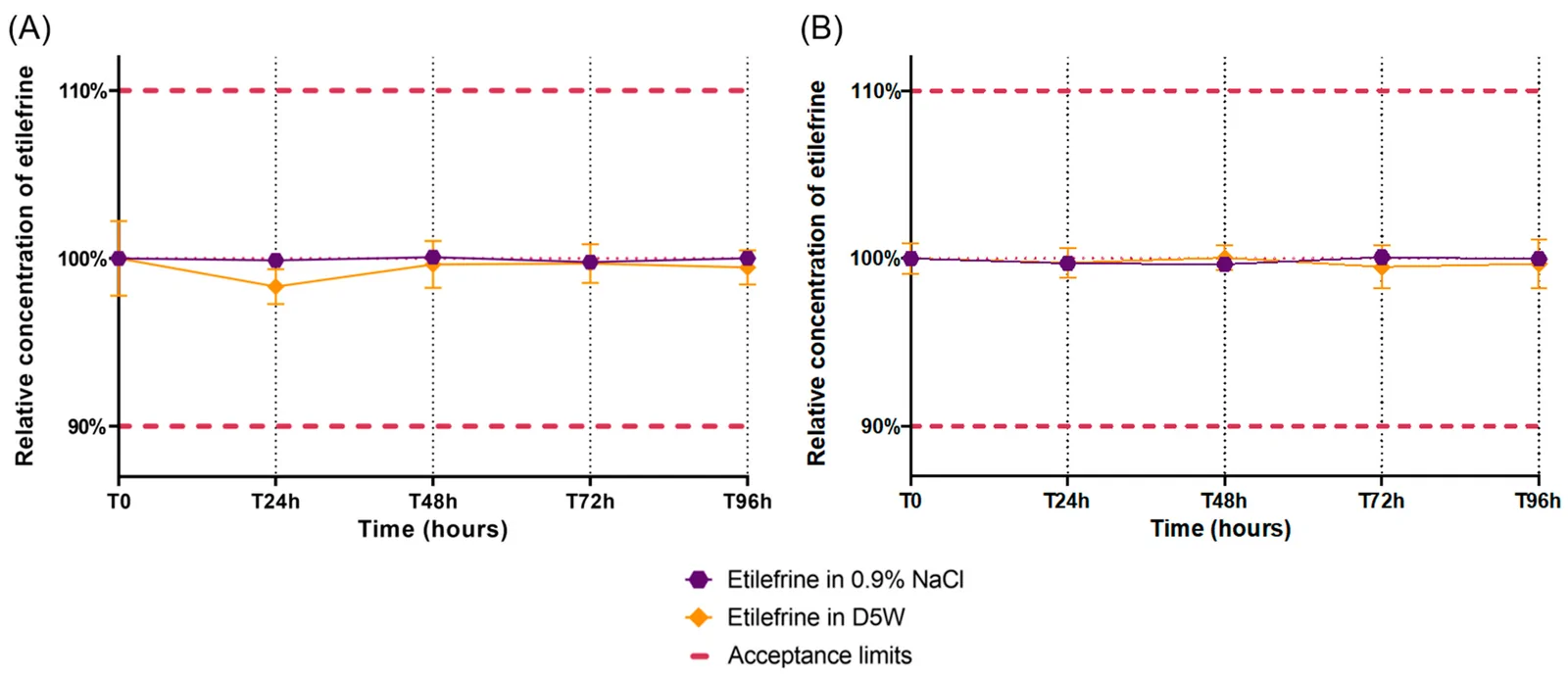
Relative concentration (Ct/C0) of etilefrine (4 µg/mL) diluted in either 0.9% NaCl or dextrose 5% in water (D5W), stored in (**A**) 10 mL syringes and (**B**) 50 mL syringes, over 96 h (*n* = 3 for each measure).

The initial etilefrine concentrations in 10 mL syringes were 3.89 ± 0.02 µg/mL (*n* = 3) and 3.95 ± 0.09 µg/mL (*n* = 3) when diluted in 0.9% NaCl and D5W, respectively. The concentration of the diluted etilefrine to 4 µg/mL remained largely within the acceptable range of 10%, as illustrated in Figure 2, when stored in 10 mL and 50 mL PP syringes at room temperature (20 ± 2 °C) for 96 h. Furthermore, no degradation products were detected.

#### 3.3.3. pH and Osmolality Assay

Key physicochemical parameters, osmolality and pH, were measured at multiple time points during the 96-h period. Both parameters remained stable throughout the 96-h storage period in the 10 mL and 50 mL syringes with both diluents, as displayed in Table 2. No statistically significant differences were observed between T0 and T96 h in either pH or osmolality under any of the conditions (*p* > 0.05).

#### 3.3.4. Non-Visible Particle Contamination Assay

**Figure 3:**
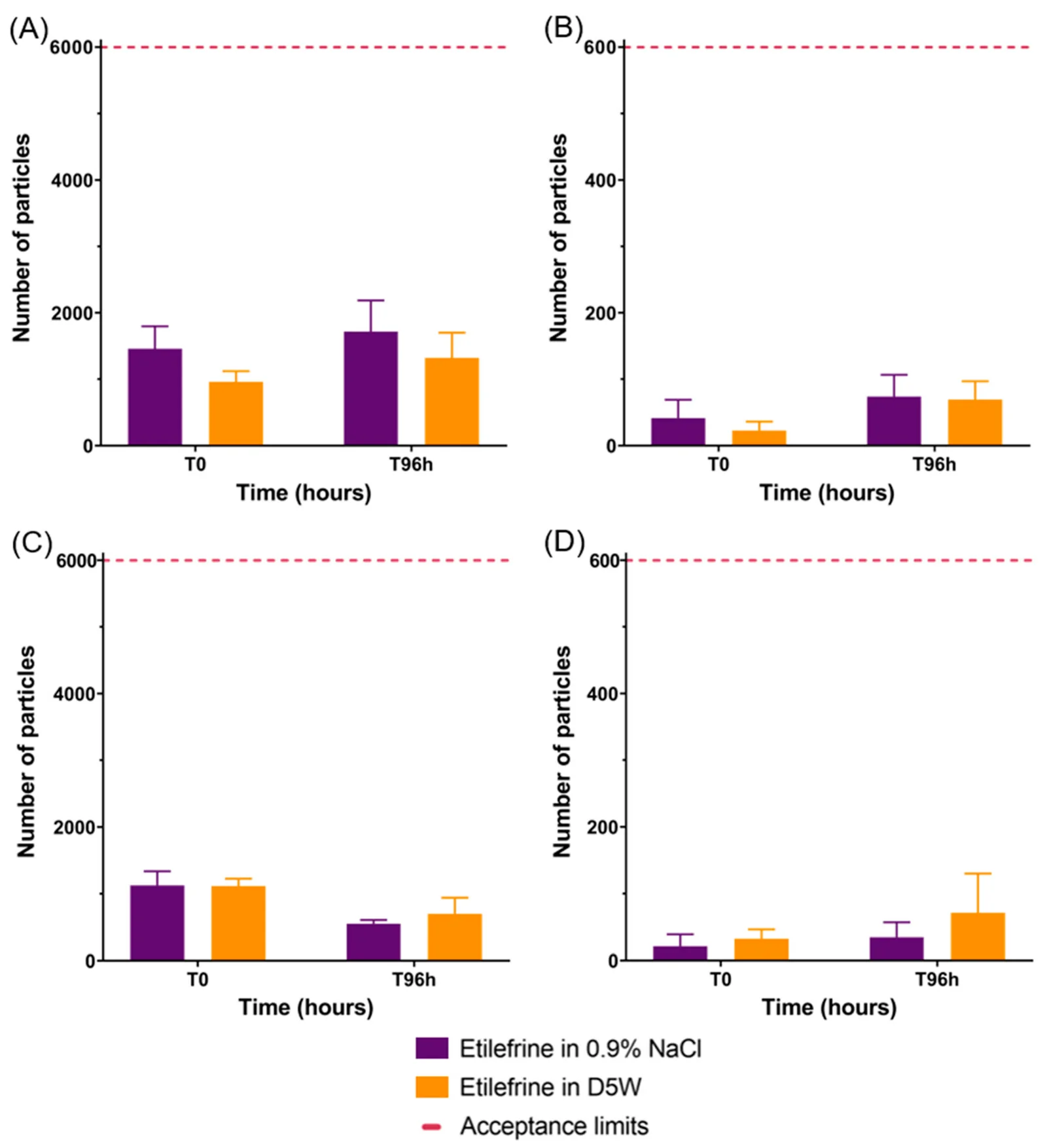
Particle count in 10 mL syringes of diluted etilefrine for (**A**) particles over 10 µm, (**B**) particles over 25 µm; particle count in 50 mL syringes of diluted etilefrine for (**C**) particles over 10 µm, (**D**) particles over 25 µm, at T0 and T96 h in both solvents, 0.9% NaCl (purple) and dextrose 5% in water (D5W) (yellow), with *n* = 9 for each time point and each solvent.

The number of particles detected both at the start and at the conclusion of the stability study was significantly below the maximum permitted limits in both syringe volumes. Specifically, particles larger than 10 µm were under the threshold of 6000 particles, and those larger than 25 µm were also far below the limit of 600 particles, as detailed in Figure 3.

## 4. Discussion

This study established that diluted etilefrine in both 0.9% NaCl and D5W solutions remained physicochemically stable for at least 96 h when stored in PP syringes at room temperature. Specifically, drug concentrations remained close to their initial values, consistently exceeding the accepted 90% threshold [12]. In addition to chemical stability, physical parameters such as osmolality and pH exhibited no relevant variations over a 96-h period, while subvisible particle counts remained consistently low and within European Pharmacopoeia limits [14]. Although particle measurements were not conducted at each time point, the lack of any visible alteration strongly suggests that no particle formation occurred throughout the study duration.

To our knowledge, this is the first study to provide stability data for diluted etilefrine preparations at a concentration relevant to neonatal chylothorax management [8,9,10]. These findings therefore address an important gap in the literature and provide practical information for clinicians and hospital pharmacists involved in the preparation and administration of etilefrine infusions.

The marked stability observed in this study aligns with the intrinsic chemical robustness of etilefrine, as previously reported by Zilker et al., who analyzed etilefrine hydrochloride ampoules (10 mg/mL) manufactured between 1949 and 1962 [17]. Using HPLC-UV, they quantified the remaining etilefrine and found that more than 98% of the initial drug content remained even after more than 40 years beyond their expiration dates. They identified a single degradation product, which, after normalization, exhibited an intensity of 0.3%. Despite the lack of information regarding excipients or storage conditions, and although their findings concern the concentrated formulation rather than diluted preparations, the long-term stability reported by Zielker et al. supports the high intrinsic stability of etilefrine. It was further demonstrated by Attia et al., who investigated the degradation behavior of etilefrine under severe stress conditions with the specific aim of generating and characterizing degradation products [18]. They reported exceptional resistance to hydrolytic degradation, with no detectable degradation after reflux under highly acidic or basic conditions. In their study, complete degradation was only achieved under severe thermal oxidative stress (reflux in 50% H_2_O_2_ for 7 h), resulting in the formation of a cyclized oxidative degradation product. These findings are consistent with our observations, as etilefrine remained essentially intact (<1% degradation) under acidic, basic, oxidative, and photolytic stress at room temperature, whereas significant degradation was observed only under severe thermal stress (80 °C for 8 to 48 h). As no degradation products or active content loss was detected under clinically relevant storage conditions, structural characterization of the thermal degradation peaks was beyond the scope of the present study. However, the successful chromatographic separation of these thermal degradation peaks from the intact drug peak without overlaps validates the stability-indicating power of our newly developed HPLC-DAD method [19]. The incorporation of sodium octanesulfonate as an ion-pairing agent at pH 3 significantly enhances the chromatographic retention and profile of protonated etilefrine [20,21]. This validates the method as a reliable analytical tool for monitoring etilefrine stability without interference from degradation products.

From a clinical standpoint, the consistently low subvisible particle counts observed are particularly relevant in neonatal intensive care settings. Neonates are especially vulnerable to particulate contamination due to their small vessel diameter, high intravenous fluid exposure relative to body weight, and extended treatment duration, which is further prolonged by continuous infusion regimens [22,23]. Intravenously infused particles may accumulate in the pulmonary capillaries, potentially leading to microvascular occlusion, thrombosis, phlebitis, and systemic inflammatory responses [22,23,24]. These concerns are especially important in neonates with refractory chylothorax, who require extended continuous infusions and often exhibit pre-existing pulmonary compromise. Although these low particle counts are reassuring, they do not substitute for the continued use of particle-retentive in-line filters currently recommended in neonatal intensive care units [24]. The physicochemical stability of diluted etilefrine has practical implications for medication management. Because physicochemical stability was maintained throughout the study period, syringe replacement may be guided primarily by microbiological standards rather than potential chemical degradation. This may reduce unnecessary manipulations and consequently limit contamination risks in this vulnerable population.

For refractory neonatal chylothorax, etilefrine is prepared at a fixed dilution (4 µg/mL) and administered via continuous intravenous infusion, with dosing adjusted via infusion rates ranging from 0.2 mL/h to more than 1 mL/h. Consequently, using syringe pumps, the drug may be delivered with syringes of different nominal volumes (10, 20, or 50 mL), depending on clinical and protocol requirements. The physicochemical stability of a drug within a syringe is primarily affected by container–content interactions and permeability to water and gas [25]. These factors are influenced by the material properties and the surface area-to-volume ratio of the container [26,27]. The fully filled 10 mL syringe used in this study exhibited the highest PP surface area-to-volume ratio, and the fully filled 50 mL syringe exhibited the highest surface contact with the elastomer plunger. The 10, 20, and 50 mL syringes share an identical design and material composition across formats [28]. Thus, our study in 10 and 50 mL syringes represents the worst-case scenarios for potential degradation. In accordance with ICH Q1D guidelines for stability study design, data obtained from 10 and 50 mL syringes are considered representative and could be extrapolated to the 20 mL syringe [26].

Despite these promising results, certain limitations must be acknowledged, including the fact that the study conditions did not fully replicate the actual neonatal intensive care administration environment. In particular, microbiological stability was not assessed, as the study was designed to characterize the physicochemical stability of the preparation. This focus aligns with the primary objective of this study, which was to address a clinical question concerning the stability of the diluted molecule in preparation for a clinical trial on etilefrine efficacy in neonates with refractory chylothorax. Consequently, as a preliminary trial support study, it was not intended to determine a microbiological shelf-life. Therefore, the 96-h study period should not be interpreted as a microbiological storage period, but rather as a timeframe allowing the physicochemical stability of the etilefrine preparation to be assessed.

Additionally, we only evaluated a single concentration of 4 µg/mL, whereas more premature infants with very low body weight may require more diluted preparations to align with infusion rates ranging from 0.2 to 1 µg/kg/h. Finally, as critically ill neonates often have very limited vascular access, frequent co-infusion of drugs and parenteral nutrition through shared lines may generate particulate matter and compromise treatment safety due to physicochemical incompatibilities [29,30]. Y-site compatibility with other drugs or parenteral nutrition remains to be determined [31].

## 5. Conclusions

This study established a solid, evidence-based foundation for the safe preparation of highly diluted etilefrine infusions for neonatal and pediatric care. By resolving empirical compounding practices, these findings directly optimize clinical workflows and medication safety in a highly vulnerable population. Ultimately, securing this practical pharmaceutical step serves as a prerequisite that paves the way for future controlled clinical trials to formally evaluate the efficacy and pharmacokinetics of etilefrine in the management of refractory neonatal chylothorax.

## Figures and Tables

**Table 1:** Forced degradation conditions applied to etilefrine to determine the stability-indicating capacity of the developed HPLC-DAD quantification method.

Degradation Condition	Condition *	Duration for Etilefrine in 0.9% NaCl	Duration for Etilefrine in D5W
Heat	80 °C	48 h	8 h
Acidic	5 M HCl	48 h	48 h
Basic	5 M NaOH	48 h	48 h
Oxidative	35% H_2_O_2_	48 h	48 h
Photodegradation	UV light exposure	48 h	48 h

* All experiments were performed at room temperature (20 ± 2 °C), unless mentioned otherwise.

**Table 2:** Osmolality and pH of the etilefrine solutions diluted to 4 µg/mL in either 10 mL or 50 mL syringes with 0.9% NaCl or dextrose 5% in water (D5W) over 96 h; *n* = 3 for each measure.

Syringe Volume	Time	Osmolality (mOsm/kg)		pH	
		0.9% NaCl	D5W	0.9% NaCl	D5W
10 mL	T0	284.3 ± 0.6	292.7 ± 1.2	5.79 ± 0.02	4.47 ± 0.01
	T24 h	284.7 ± 1.2	292.3 ± 2.5	5.78 ± 0.02	4.50 ± 0.01
	T48 h	285.0 ± 1.0	292.3 ± 2.3	5.78 ± 0.02	4.49 ± 0.01
	T72 h	285.3 ± 1.5	294.3 ± 0.6	5.77 ± 0.01	4.47 ± 0.01
	T96 h	285.3 ± 1.2	294.0 ± 1.0	5.76 ± 0.01	4.49 ± 0.01
50 mL	T0	279.7 ± 0.6	293.0 ± 1.0	5.97 ± 0.02	4.53 ± 0.04
	T24 h	279.3 ± 0.6	292.7 ± 1.2	5.98 ± 0.02	4.51 ± 0.03
	T48 h	278.7 ± 0.6	291.7 ± 1.5	5.94 ± 0.04	4.52 ± 0.05
	T72 h	279.7 ± 0.6	292.7 ± 0.6	5.97 ± 0.02	4.53 ± 0.05
	T96 h	279.7 ± 0.6	291.3 ± 0.6	5.96 ± 0.03	4.52 ± 0.01

## Data Availability

The data supporting the findings of this study are included in this published article and its Supplementary Materials. Detailed data underlying the statistical analyses are available from the corresponding author upon request.

## Supplementary Materials

The following supporting information can be downloaded at https://www.mdpi.com/article/10.3390/pharmaceutics18091135/s1, Figure S1: Calibration curves of the HPLC-UV method for etilefrine quantification in (A) 0.9% NaCl and (B) dextrose 5% in water (D5W), with 95% confidence intervals; Figure S2: Accuracy profiles of the validated HPLC-UV method for etilefrine quantification in (A) in 0.9% NaCl and (B) dextrose 5% in water (D5W); Figure S3: Precision of the validated HPLC-UV method for etilefrine quantification in (A) in 0.9% NaCl and (B) dextrose 5% in water (D5W); Table S1: Validation parameters of the HPLC-UV method for etilefrine quantification in 0.9% NaCl and dextrose 5% in water, (D5W).

## References

[1] Bellini C., Ergaz Z., Boccardo F., Bellini T., Campisi C.C., Bonioli E., Ramenghi L.A. (2013). Dynamics of Pleural Fluid Effusion and Chylothorax in the Fetus and Newborn: Role of the Lymphatic System. Lymphology.

[2] Bhatnagar M., Fisher A., Ramsaroop S., Carter A., Pippard B. (2024). Chylothorax: Pathophysiology, Diagnosis, and Management—A Comprehensive Review. J. Thorac. Dis..

[3] Resch B., Sever Yildiz G., Reiterer F. (2022). Congenital Chylothorax of the Newborn: A Systematic Analysis of Published Cases between 1990 and 2018. Respiration.

[4] Rocha G., Arnet V., Soares P., Gomes A.C., Costa S., Guerra P., Casanova J., Azevedo I. (2021). Chylothorax in the Neonate—A Stepwise Approach Algorithm. Pediatr. Pulmonol..

[5] Walsh M., Tutor J.D. (2025). The Management of Neonatal Chylothorax. Curr. Treat. Options Pediatr..

[6] Bellini C., Cabano R., De Angelis L.C., Bellini T., Calevo M.G., Gandullia P., Ramenghi L.A. (2018). Octreotide for Congenital and Acquired Chylothorax in Newborns: A Systematic Review. J. Paediatr. Child Health.

[7] Ohkura Y., Ueno M., Iizuka T., Udagawa H. (2018). Effectiveness of Etilefrine Regimen for Chylothorax after Esophagectomy with Thoracic Duct Resection. Esophagus.

[8] Muniz G., Hidalgo-Campos J., Valdivia-Tapia M.d.C., Shaikh N., Carreazo N.Y. (2018). Successful Management of Chylothorax With Etilefrine: Case Report in 2 Pediatric Patients. Pediatrics.

[9] Gilliot S., Le Duc K., Chopinet C., Décaudin B., Odou P., Storme L., Riadh Boukhris M. (2025). Etilefrine: A Novel Treatment for Refractory, Life-Threatening Chylothorax in the Newborn. Pediatrics.

[10] Tomobe Y., Mizuguchi U., Shimotakahara A., Shimojima N., Okazaki K. (2020). Combination Therapy with Etilefrine and Pleurodesis for Refractory Congenital Chylothorax. Biomed. Hub.

[11] Guillem P., Billeret V., Lecomte Houcke M., Triboulet J.P. (1999). Successful Management of Post-Esophagectomy Chylothorax/Chyloperitoneum by Etilefrine. Dis. Esophagus.

[12] SFPC, GERPAC (2013). Methodological Guidelines for Stability Studies of Hospital Pharmaceutical Preparations.

[13] European Medicines Agency (2003). Q 1 A (R2) Stability Testing of New Drug Substances and Products.

[14] 2.9.19. Particulate Contamination: Sub-Visible Particles-Pharmacopée Européenne En Ligne. https://pheur-online.edqm.eu/content/20919/en/current/?q=particule&status=A&status=N.

[15] Hubert P., Nguyen-Huu J.-J., Boulanger B., Chapuzet E., Cohen N., Compagnon P.-A., Dewé W., Feinberg M., Laurentie M., Mercier N. (2007). Harmonization of Strategies for the Validation of Quantitative Analytical Procedures: A SFSTP Proposal Part III. J. Pharm. Biomed. Anal..

[16] Hubert P., Nguyenhuu J., Boulanger B., Chapuzet E., Chiap P., Cohen N., Compagnon P., Dewe W., Feinberg M., Lallier M. (2004). Harmonization of Strategies for the Validation of Quantitative Analytical Procedures: A SFSTP Proposal Part I. J. Pharm. Biomed. Anal..

[17] Zilker M., Sörgel F., Holzgrabe U. (2018). A Stability-Study of Expired Ampoules Manufactured More than 40 Years Ago. J. Pharm. Biomed. Anal..

[18] Attia K.A.M., El-Abasawy N.M., El-Olemy A., Abdel-Raoof A.M. (2016). High Performance Liquid Chromatography Based on Computational Study for the Determination of Etilefrine Hydrochloride in the Presence of Its Oxidative Degradation Product. Anal. Chem. Lett..

[19] Singh S., Junwal M., Modhe G., Tiwari H., Kurmi M., Parashar N., Sidduri P. (2013). Forced Degradation Studies to Assess the Stability of Drugs and Products. TrAC Trends Anal. Chem..

[20] Bidlingmeyer B.A. (1980). Separation of Ionic Compounds by Reversed-Phase Liquid Chromatography An Update of Ion-Pairing Techniques. J. Chromatogr. Sci..

[21] Dai J., Carr P.W. (2005). Role of Ion Pairing in Anionic Additive Effects on the Separation of Cationic Drugs in Reversed-Phase Liquid Chromatography. J. Chromatogr. A.

[22] Perez M., Maiguy-Foinard A., Barthélémy C., Décaudin B., Odou P. (2016). Particulate Matter in Injectable Drugs: Evaluation of Risks to Patients. Pharm. Technol. Hosp. Pharm..

[23] Benlabed M., Martin Mena A., Gaudy R., Perez M., Genay S., Hecq J.-D., Odou P., Lebuffe G., Décaudin B. (2018). Analysis of Particulate Exposure during Continuous Drug Infusion in Critically Ill Adult Patients: A Preliminary Proof-of-Concept in Vitro Study. Intensive Care Med. Exp..

[24] Martin Mena A., Masse M., Négrier L., Nguyen T.H., Ladam B., Storme L., Barthélémy C., Odou P., Genay S., Décaudin B. (2021). Optimising an Infusion Protocol Containing Cefepime to Limit Particulate Load to Newborns in a Neonatal Intensive Care Unit. Pharmaceutics.

[25] Sacha G.A., Saffell-Clemmer W., Abram K., Akers M.J. (2010). Practical Fundamentals of Glass, Rubber, and Plastic Sterile Packaging Systems. Pharm. Dev. Technol..

[26] Munden R.P. (2017). ICH Q1D-Bracketing and Matrixing Designs for Stability Testing of Drug Substances and Drug Products. ICH Quality Guidelines.

[27] Jenke D.R., Rabinow B.E. (2017). Proper Accounting for Surface Area to Solution Volume Ratios in Exaggerated Extractions. PDA J. Pharm. Sci. Technol..

[28] Becton Dickinson and Company (2024). Technical Data Sheet V201-009 Rev08, BD Plastipak Syringes Without Needles and BD Hypodermic Syringes Without Needle. https://digitalassets.avantorsciences.com/adaptivemedia/rendition?id=d0c04e607bdd14bb792738e385d4b7bd7973aa32&vid=d0c04e607bdd14bb792738e385d4b7bd7973aa32&prid=original&clid=SAPDAM.

[29] Benlabed M., Perez M., Gaudy R., Genay S., Lannoy D., Barthélémy C., Odou P., Lebuffe G., Décaudin B. (2019). Clinical Implications of Intravenous Drug Incompatibilities in Critically Ill Patients. Anaesth. Crit. Care Pain Med..

[30] Foinard A., Décaudin B., Barthélémy C., Debaene B., Odou P. (2012). Impact of Physical Incompatibility on Drug Mass Flow Rates: Example of Furosemide-Midazolam Incompatibility. Ann. Intensive Care.

[31] Foinard A., Perez M., Décaudin B., Barthélémy C., Tournoys A., Storme L., Odou P. (2014). Heparin Stability in Parenteral Nutrition Bags Prepared in a Neonatal ICU. Crit. Care.

