# Supplementary material for: Diluted Etilefrine for Neonatal Chylothorax: A 96-Hour Physicochemical Stability Study
Source: Pharmaceutics. 2026 Sep 9;18(9):1135. doi: 10.3390/pharmaceutics18091135 (PMC13610745; doi:10.3390/pharmaceutics18091135)
Supplement: Supplementary file 1 [file pharmaceutics-18-01135-s001.zip › pharmaceutics-4542473-supplementary.pdf]

Diluted etilefrine for neonatal chylothorax: a 96-hour physicochemical stability study

Louisa Bourel[1], Sixtine Gilliot[1], Zeina Boulos[1], Yousra Alouadni[1], Kévin Le Duc[2,3], Mohamed Riadh Boukhris[3], Laurent Storme[2], Anthony Martin Mena[1] and Pascal Odou[1]

[1]Univ. Lille, CHU Lille, ULR 7365 - GRITA - Groupe de Recherche sur les formes Injectables et les Technologies Associées, F-59000 Lille, France

[2] Univ. Lille, CHU Lille, ULR 2694 METRICS: Evaluation des technologies de santé et des pratiques médicales - Perinatal Environment and Health, F-59000 Lille, France

[3] CHU Lille, Department of Neonatology, Jeanne de Flandre Hospital, F-59000 Lille, France

Table of Figures

Figure S1. Calibration curves of the HPLC-UV method for etilefrine quantification in A) 0.9% NaCl and B) dextrose 5% in water (D5W), with 95% confidence intervals 3

Figure S2. Accuracy profiles of the validated HPLC-UV method for etilefrine quantification in A) in 0.9% NaCl and B) dextrose 5% in water (D5W) 4

Figure S3. Precision of the validated HPLC-UV method for etilefrine quantification in A) in 0.9% NaCl and B) dextrose 5% in water (D5W) 5

Table S1. Validation parameters of the HPLC-UV method for etilefrine quantification in 0.9% NaCl and dextrose 5% in water, (D5W) 6

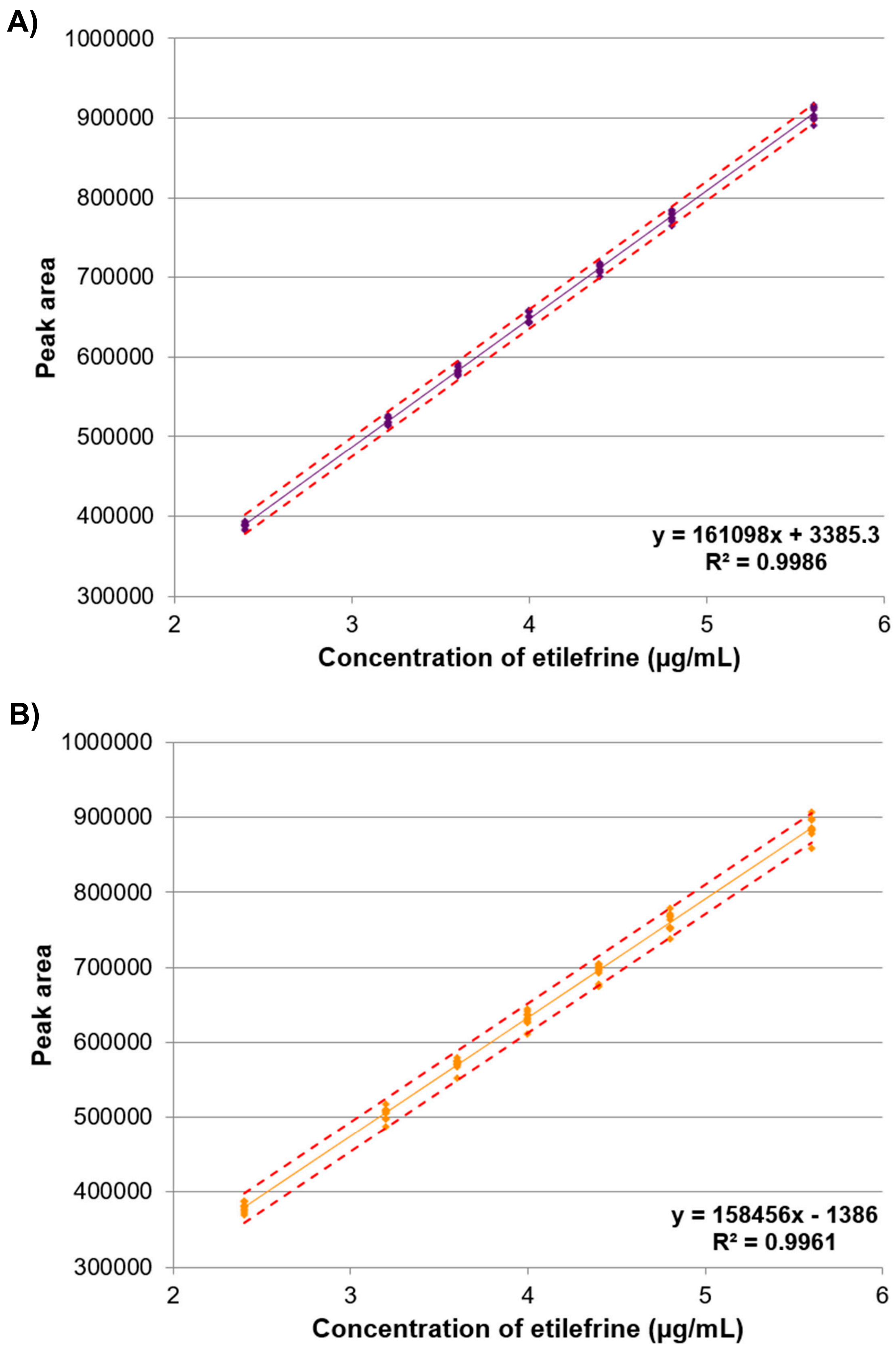

Figure S1. Calibration curves of the HPLC-UV method for etilefrine quantification in A) 0.9% NaCl and B) dextrose 5% in water (D5W), with 95% confidence intervals

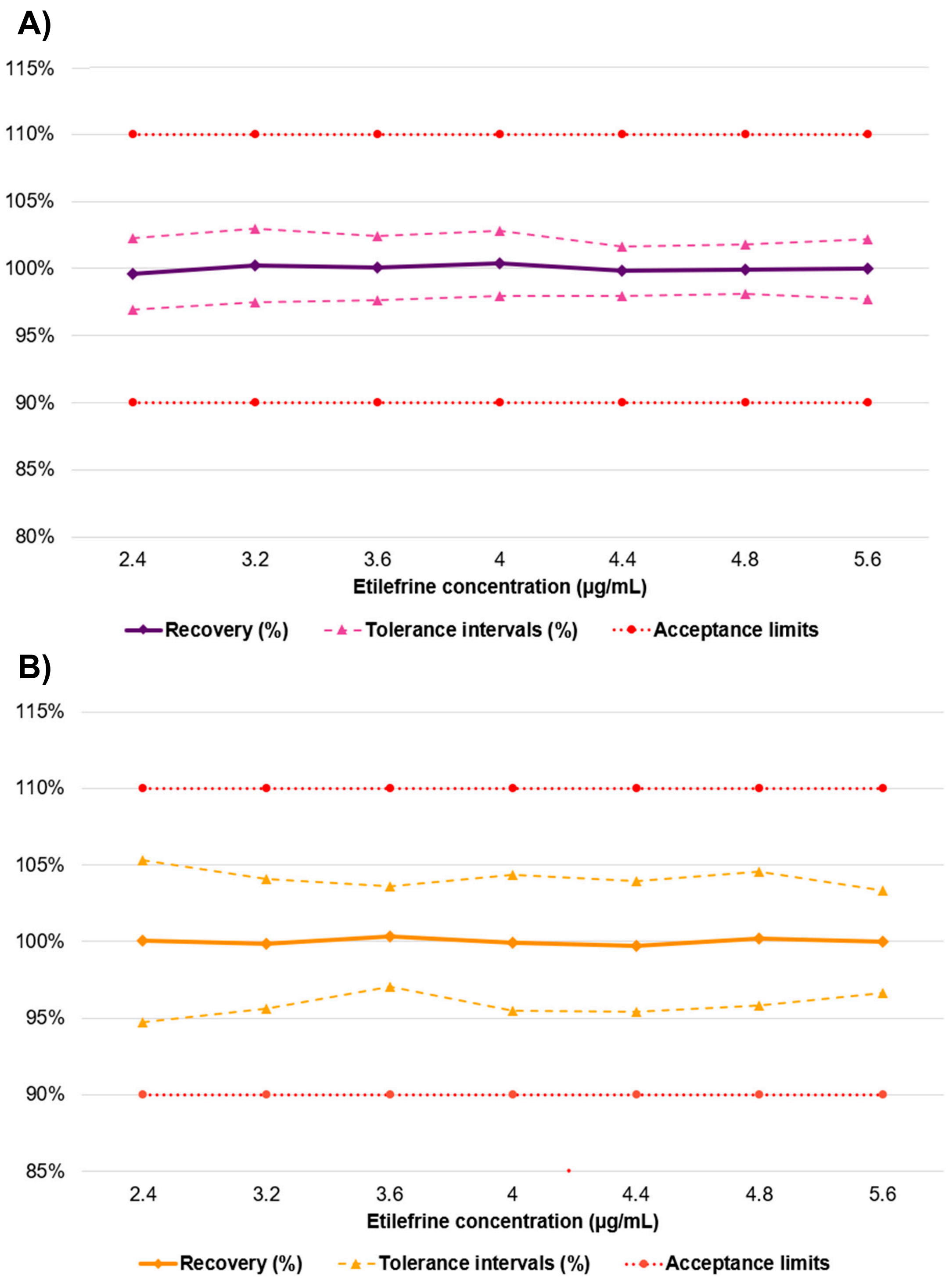

Figure S2. Accuracy profiles of the validated HPLC-UV method for etilefrine quantification in A) in 0.9% NaCl and B) dextrose 5% in water (D5W)

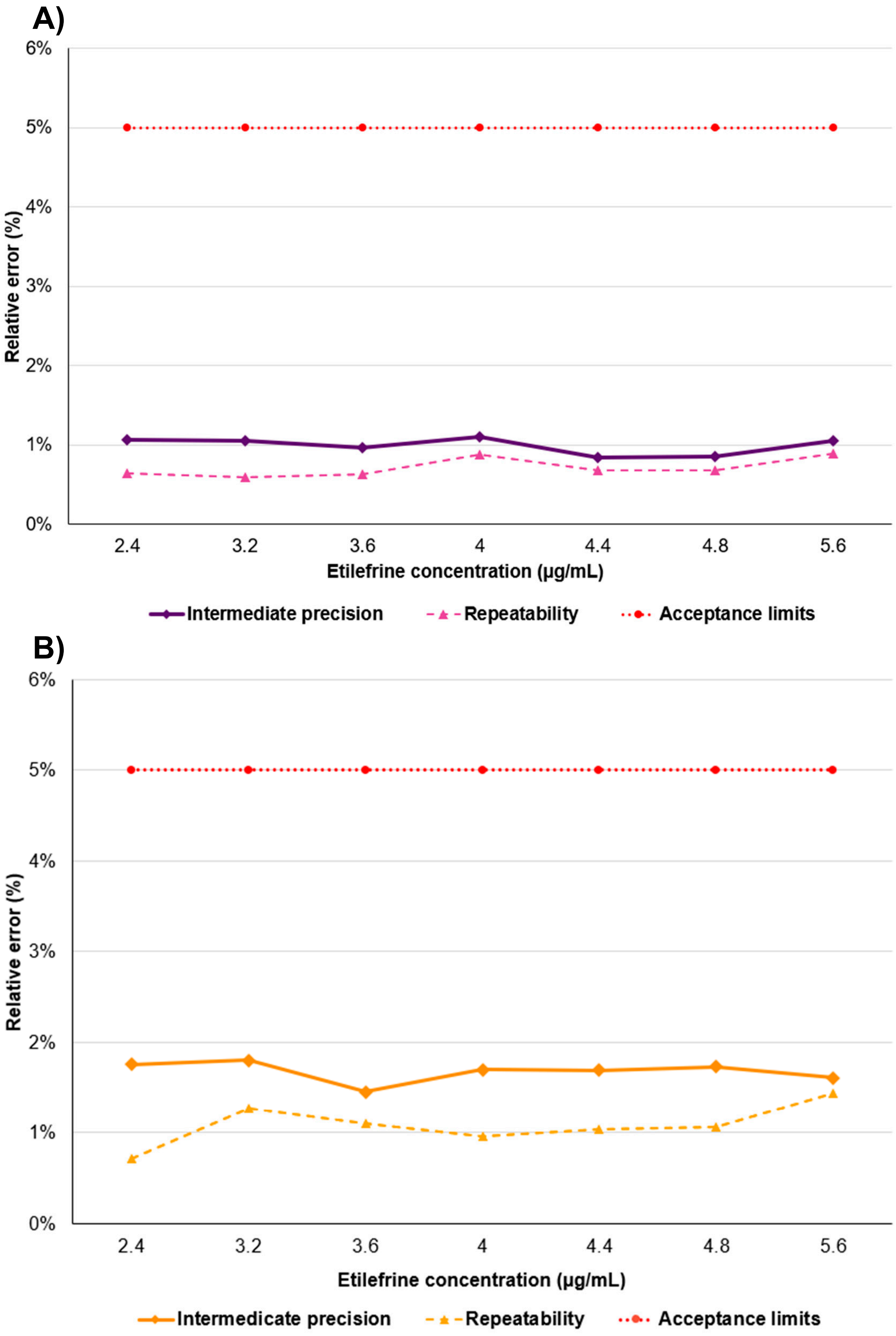

Figure S3. Precision of the validated HPLC-UV method for etilefrine quantification in A) in 0.9% NaCl and B) dextrose 5% in water (D5W)

Table S1. Validation parameters of the HPLC-UV method for etilefrine quantification in 0.9% NaCl and dextrose 5% in water, (D5W)

Diluent used	Etilefrine concentration (µg/mL)	Bias (%)	Intermediate precision (%)	Tolerance intervals (%)		Total error (%)
Etilefrine in 0.9% NaCl	2.4	-0.39	1.06	96.93	102.29	1.45
	3.2	0.21	1.05	97.48	102.94	1.25
	3.6	0.04	0.97	97.66	102.42	1.01
	4	0.35	1.10	97.93	102.78	1.45
	4.4	-0.17	0.84	97.98	101.67	1.02
	4.8	-0.06	0.85	98.08	101.80	0.91
	5.6	-0.04	1.05	97.73	102.18	1.09
Etilefrine in D5W	2.4	0.03	1.76	94.77	105.30	1.79
	3.2	-0.17	1.80	95.61	104.05	1.97
	3.6	0.33	1.45	97.04	103.62	1.79
	4	-0.07	1.70	95.51	104.36	1.77
	4.4	-0.32	1.69	95.42	103.95	2.01
	4.8	0.19	1.73	95.83	104.55	1.92
	5.6	0.00	1.61	96.67	103.33	1.61
